# Supplementary material for: Changes over time in the health and functioning of older people moving into care homes: analysis of data from the English Longitudinal Study of Ageing
Source: Age Ageing. Author manuscript; Available in PMC 2017 Sep 8. (PMC5590718; doi:10.1093/ageing/afx046)
Supplement: Supplementary information [file NIHMS73563-supplement-Supplementary_information.docx]

**APPENDIX - SUPPLEMENTAL MATERIAL**

| **Supplemental table 1**: The relationship between wave of entry to a care home and number of health conditions and level of functional deficit in ELSA participants: results from a linear regression model | | | | | | | | | |
| --- | --- | --- | --- | --- | --- | --- | --- | --- | --- |
|  | Outcome* | R^2^ | F | df | p (model) | t (wave) | p (wave) | Coefficient (wave) | [95% CI] |
| Number of health conditions and level of functional deficit | | 0.05 | 4.47 | 3,250 | 0.0045 | 3.30 | 0.0011 | 0.12 | [0.05-0.19] |
| *Adjusted for wave and age. CI confidence interval | | | | | |  |  |  |  |

| **Supplemental table 2**: Associations between health conditions and time (wave of entry to a care home) in ELSA participants: results from logistic regression models | | | | | |
| --- | --- | --- | --- | --- | --- |
|  | Outcome | Odds ratio (wave)* | [95% CI] | p |  |
| High blood pressure | | 1.33 | [1.14-1.56] | 0.0003 |  |
| Memory problems | | 1.29 | [1.08-1.55] | 0.0052 |  |
| Diabetes |  | 1.29 | [1.05-1.59] | 0.0147 |  |
| Cancer |  | 1.29 | [0.99-1.72] | 0.06 |  |
| Lung disease | | 1.18 | [0.84-1.70] | 0.35 |  |
| Heart disease | | 1.14 | [0.98-1.33] | 0.09 |  |
| Arthritis |  | 1.13 | [0.97-1.31] | 0.11 |  |
| Stroke |  | 1.02 | [0.86-1.22] | 0.79 |  |
| Poor self-rated health | | 0.99 | [0.83-1.18] | 0.93 |  |
| *Adjusted for age. CI confidence interval | | | | | |

| **Supplemental table 3**: The relationship between wave of entry to a care home and number of IADL and ADL deficits in ELSA participants about to enter a care home: results from linear regression models | | | | | | | | | |
| --- | --- | --- | --- | --- | --- | --- | --- | --- | --- |
|  | Outcome* | R^2^ | F | df | p (model) | t (wave) | p (wave) | Coefficient (wave) | [95% CI] |
| Number IADL | | 0.03 | 3.59 | 2,247 | 0.0291 | 2.68 | 0.0080 | 0.09 | [0.02-0.16] |
| Number ADL | | 0.00 | 0.13 | 2,247 | 0.88 | 0.48 | 0.63 | 0.01 | [-0.04-0.07] |
| *Adjusted for wave and age. CI confidence interval | | | | | |  |  |  |  |

| **Supplemental table 4**: Associations between baseline age, wave and sex in ELSA participants about to enter a care home: results from two-way ANOVA | | | | |
| --- | --- | --- | --- | --- |
|  | | | | |
|  | Predictor* | F | df | p |
| Wave |  | 25.70 | 1,250 | <0.0001 |
| Sex | | 18.69 | 1,250 | <0.0001 |
| Wave*sex interaction | | 0.19 | 1,250 | 0.66 |
| *Age as outcome. | | | | |
